# Piezo-Assisted Gap Arthroplasty: A Safer Surgical Approach for Temporomandibular Joint Ankylosis

**DOI:** 10.7759/cureus.113275

**Published:** 2026-07-24

**Authors:** Kshitij Bang, Onkar Redekar, Ramakrishna Shenoi, Pranav Ingole, Anant Kahare

**Affiliations:** 1 Oral and Maxillofacial Surgery, Vidya Shikshan Prasarak Mandal’s Dental College and Research Centre, Nagpur, IND

**Keywords:** bony ankylosis, buccal fat pad, coronoidectomy, gap arthroplasty, interposition arthroplasty, pediatric tmj ankylosis, piezoelectric surgery, trismus

## Abstract

Temporomandibular joint ankylosis (TMJa) is an incapacitating disease due to the fusion of the mandibular condyle with the temporal bone, leading to restricted mouth opening. In children, early surgical intervention is required to avoid alterations in growth and irreversible facial deformity. This case report outlines the treatment of unilateral Type III bony TMJa with piezo-assisted gap arthroplasty and pedicled buccal fat pad interposition in a six-year-old female. We performed osteotomy with a piezoelectric surgical device to control bone resection and decrease the potential for injury to surrounding soft tissues and vital structures. Incision was made approximately 10 mm apart, and ipsilateral coronoidectomy of the mandible was performed to increase range of motion. Early physiotherapy was performed postoperatively to maintain functional mouth opening. The patient showed more than 20 mm mouth opening when followed up, with good functional recovery. The piezoelectric surgical technique offers an accurate, secure modern approach for the surgical management of TMJa compared to the conventional rotary instruments.

## Introduction

Temporomandibular joint ankylosis (TMJa) is a disabling craniomaxillofacial condition resulting from fibrous or bony fusion of the mandibular condyle with the temporal bone, leading to reduced mandibular movement and impaired facial growth [1]. The condition predominantly affects children and adolescents. The underlying pathophysiology involves intra-articular hemorrhage or inflammation following trauma or infection, which promotes fibrosis and subsequent heterotopic bone formation across the joint space. Progressive ossification ultimately restricts condylar movement and alters normal mandibular growth, making early diagnosis and surgical intervention essential to restore function and prevent irreversible craniofacial deformity [2]. Untreated ankylosis in pediatric patients may result in mandibular hypoplasia, facial asymmetry, malocclusion, compromised airway function, and significant psychosocial distress [3].

Several surgical approaches have been described for the management of TMJa, including gap arthroplasty, interpositional arthroplasty, and joint reconstruction. Gap arthroplasty remains a widely used technique; however, recurrence rates reported range from 2.56% to 11.9% depending on surgical technique and postoperative physiotherapy compliance [4]. Piezoelectric surgery has emerged as an alternative to conventional rotary instruments. The technique selectively cuts mineralized tissues and prevents injury to adjacent soft tissues and important neurovascular structures [5].

This report describes the successful management of unilateral Sawhney’s Type III bony TMJa (bony bridge between the mandibular ramus and the zygomatic arch) in a pediatric patient using piezo-assisted gap arthroplasty with autogenous interpositional grafting.

## Case presentation

Patient information

A six-year-old female presented with the chief complaint of reduced mouth opening from the age of two years, which had slowly reduced to 2 mm at present. The guardian reported difficulty with feeding, and she was only able to tolerate a liquid diet. There was a history of trauma due to a fall while playing at home at the age of two years. No history of systemic illness or previous surgical intervention was reported.

Clinical findings

**Figure 1:**
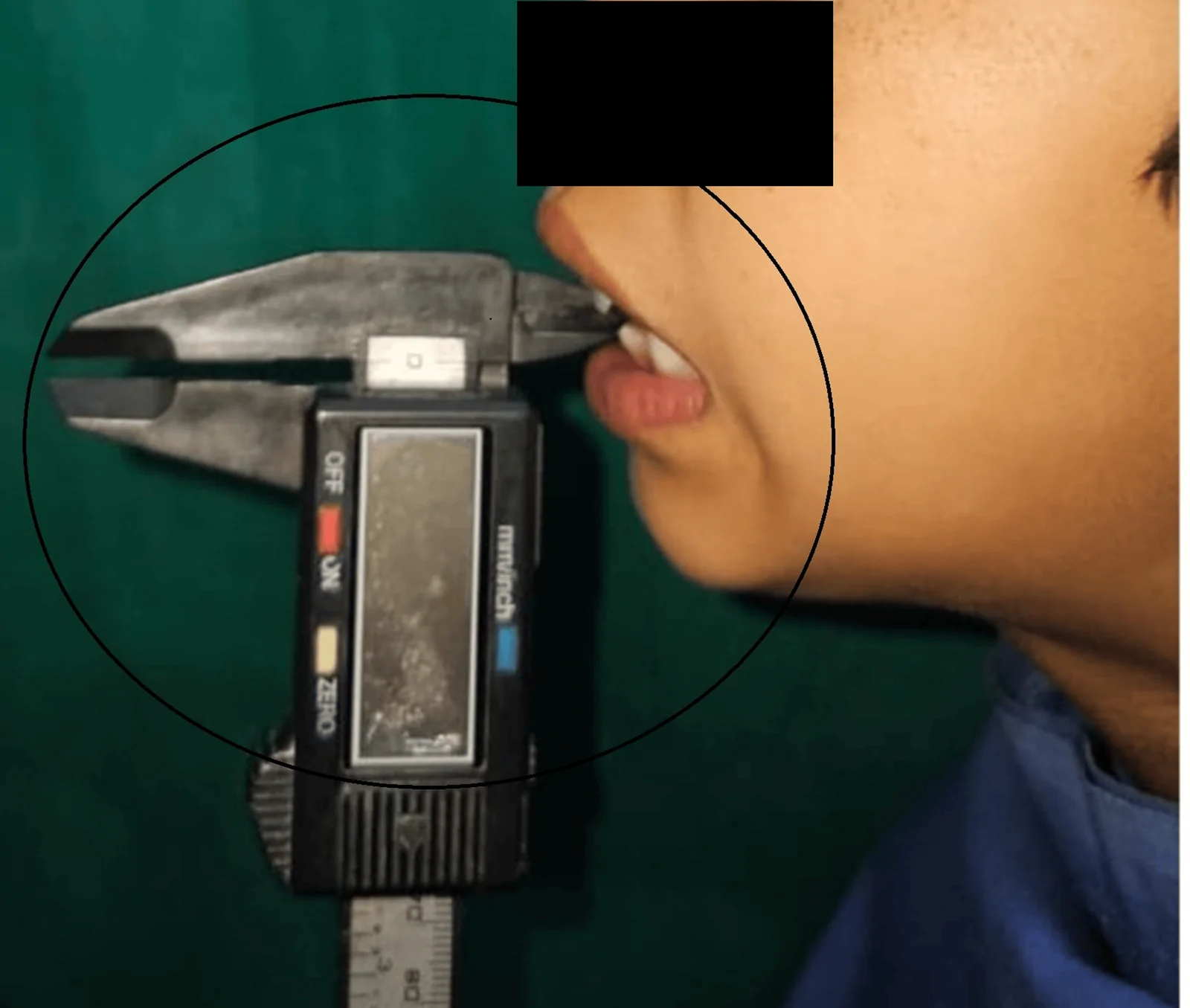
Preoperative interincisal mouth opening of approximately 2 mm measured using a Vernier caliper (black circle).

Extraoral examination revealed facial asymmetry, fullness of the face on the right side, which was the affected side, flatness of the face on the left side, which was the non-affected side, and chin deviation on the right side. Facial profile was convex due to mandibular hypoplasia. As the interincisal mouth opening was approximately 2 mm (Figure 1), detailed intraoral examination was not possible, but occlusion looked reasonably satisfactory. Temporomandibular joint movements were not palpable on the right side, and slight movements were palpable on the left side.

Radiographic assessment

**Figure 2:**
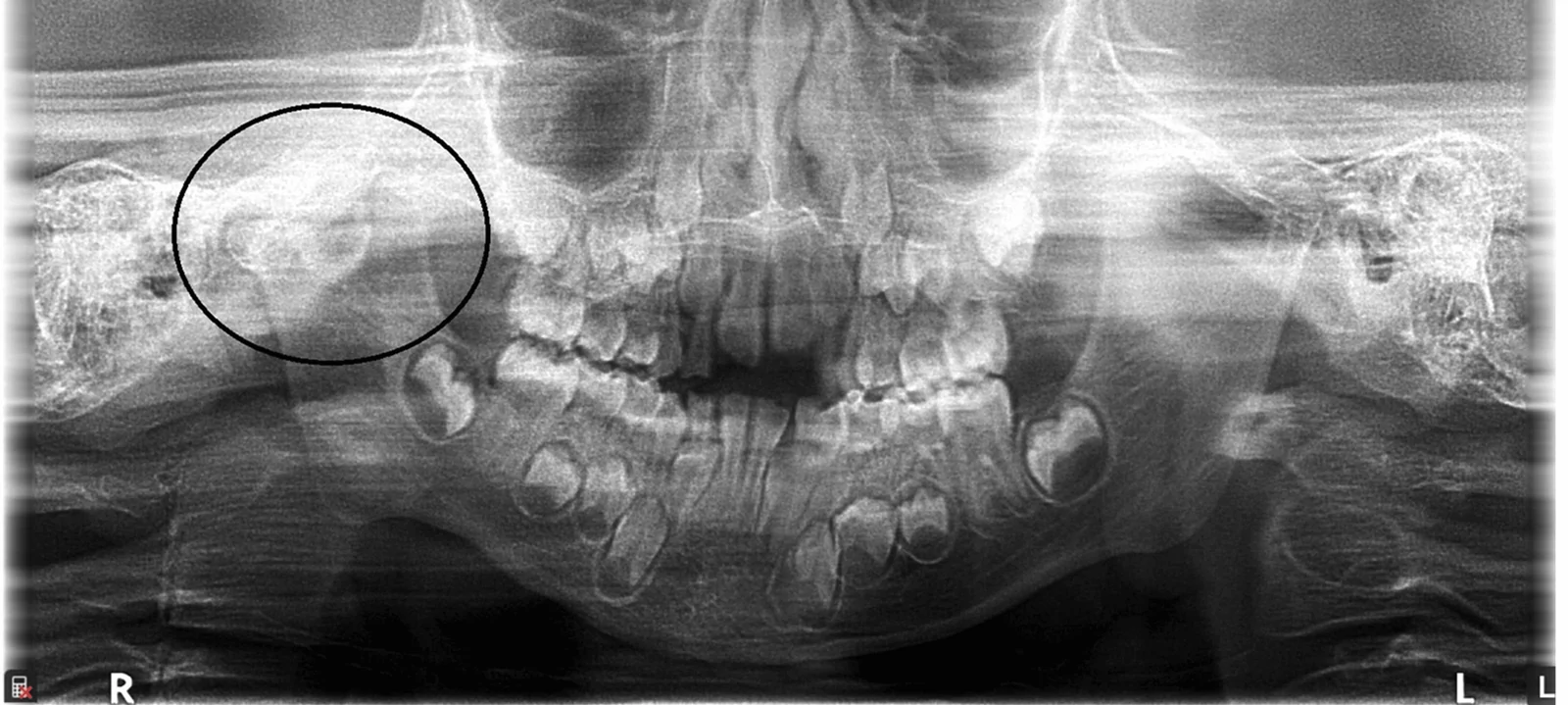
Preoperative orthopantomogram showing right temporomandibular joint bony ankylosis with obliteration of the joint space and fusion of the mandibular condyle to the temporal bone (black circle).

**Figure 3:**
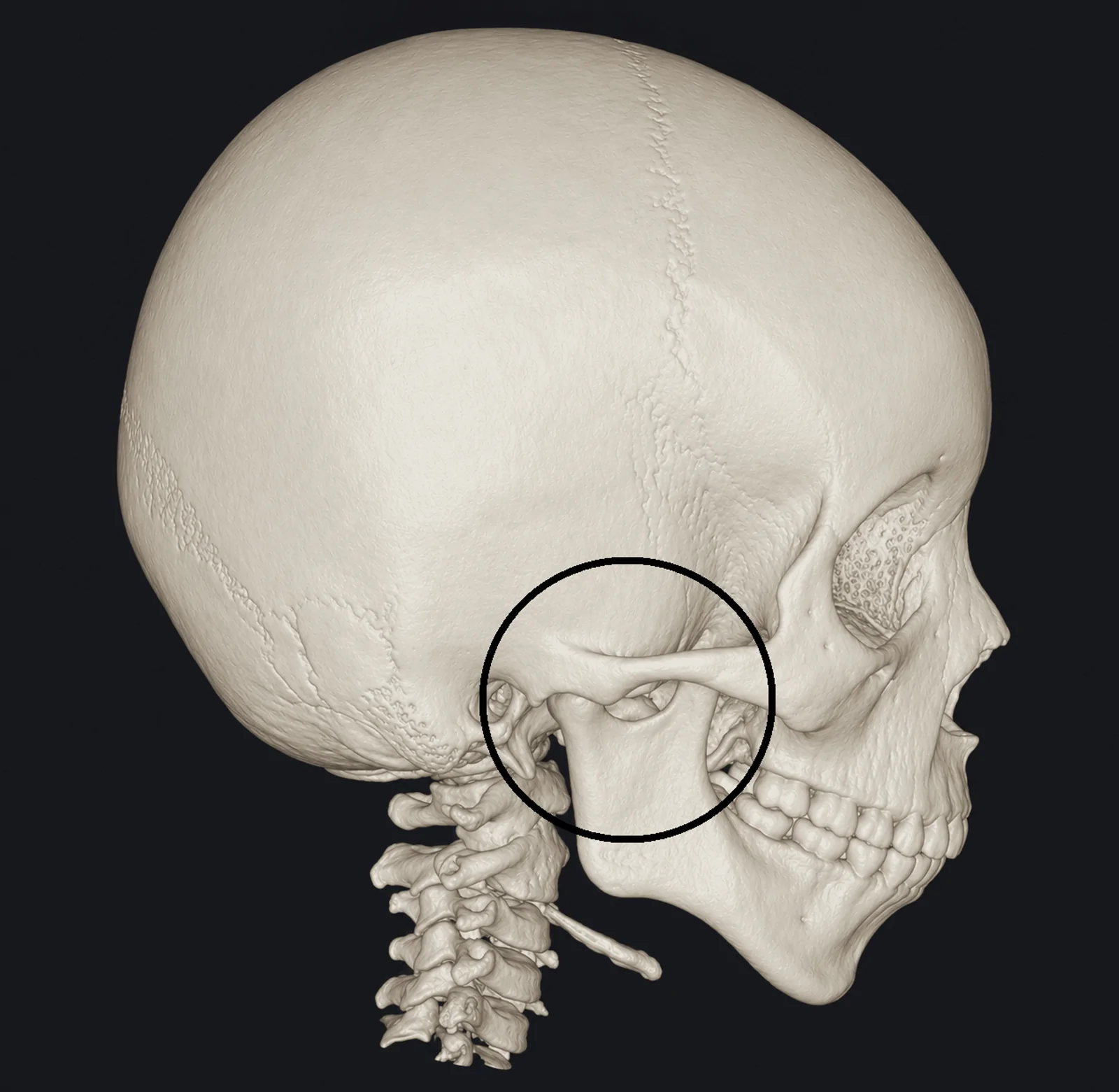
Preoperative three-dimensional computed tomography reconstruction demonstrating right temporomandibular joint bony ankylosis with fusion of the mandibular condyle to the temporal bone, resulting in obliteration of the joint space (black circle).

Preoperative orthopantomogram (OPG) showed right temporomandibular joint bony ankylosis with obliteration of the joint space and fusion of the mandibular condyle to the temporal bone (Figure 2). Preoperative three-dimensional computed tomography reconstruction demonstrated right temporomandibular joint bony ankylosis with fusion of the mandibular condyle to the temporal bone, resulting in obliteration of the joint space (Figure 3).

Based on clinical findings and radiographic features, a diagnosis of unilateral right-sided TMJa was established. According to the classification proposed by Sawhney, the condition was categorized as Type III bony TMJa as there was a bony bridge between the mandibular ramus and the zygomatic arch [6].

Surgical management

Under general anaesthesia with fiberoptic nasotracheal intubation, surgery was performed. A standard preauricular approach was used to access the temporomandibular joint. Incision was taken through the skin and subcutaneous tissue; careful blunt dissection was performed through the superficial musculoaponeurotic system with care to preserve the branches of the facial nerve, particularly the temporal branch. The temporalis fascia was identified and incised to expose the zygomatic arch and joint capsule. The periosteum was elevated to expose the ankylotic mass between the glenoid fossa and mandibular condyle.

**Figure 4:**
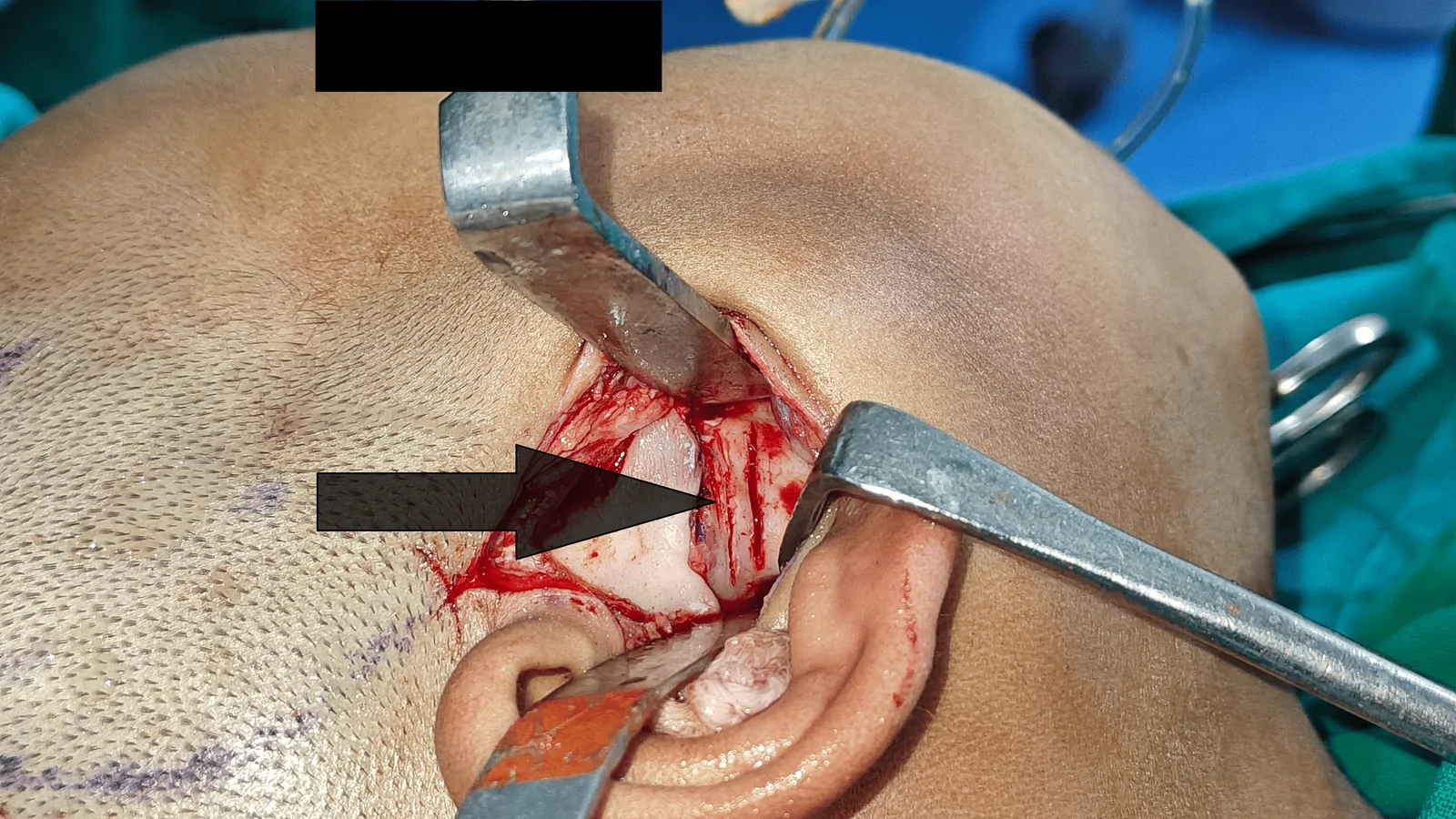
Intraoperative image of the ankylosed temporomandibular joint, with osteotomy marking performed using a piezoelectric surgical device at the planned resection site (black arrow).

Osteotomy of the ankylotic mass was performed using a piezoelectric surgical device (Figure 4) rather than conventional rotary instruments. Bone resection was performed under continuous saline irrigation to prevent thermal injury. The use of piezoelectric surgery provided controlled bone cutting and minimized the risk of injury to adjacent soft tissues and vascular structures such as the internal maxillary artery.

**Figure 5:**
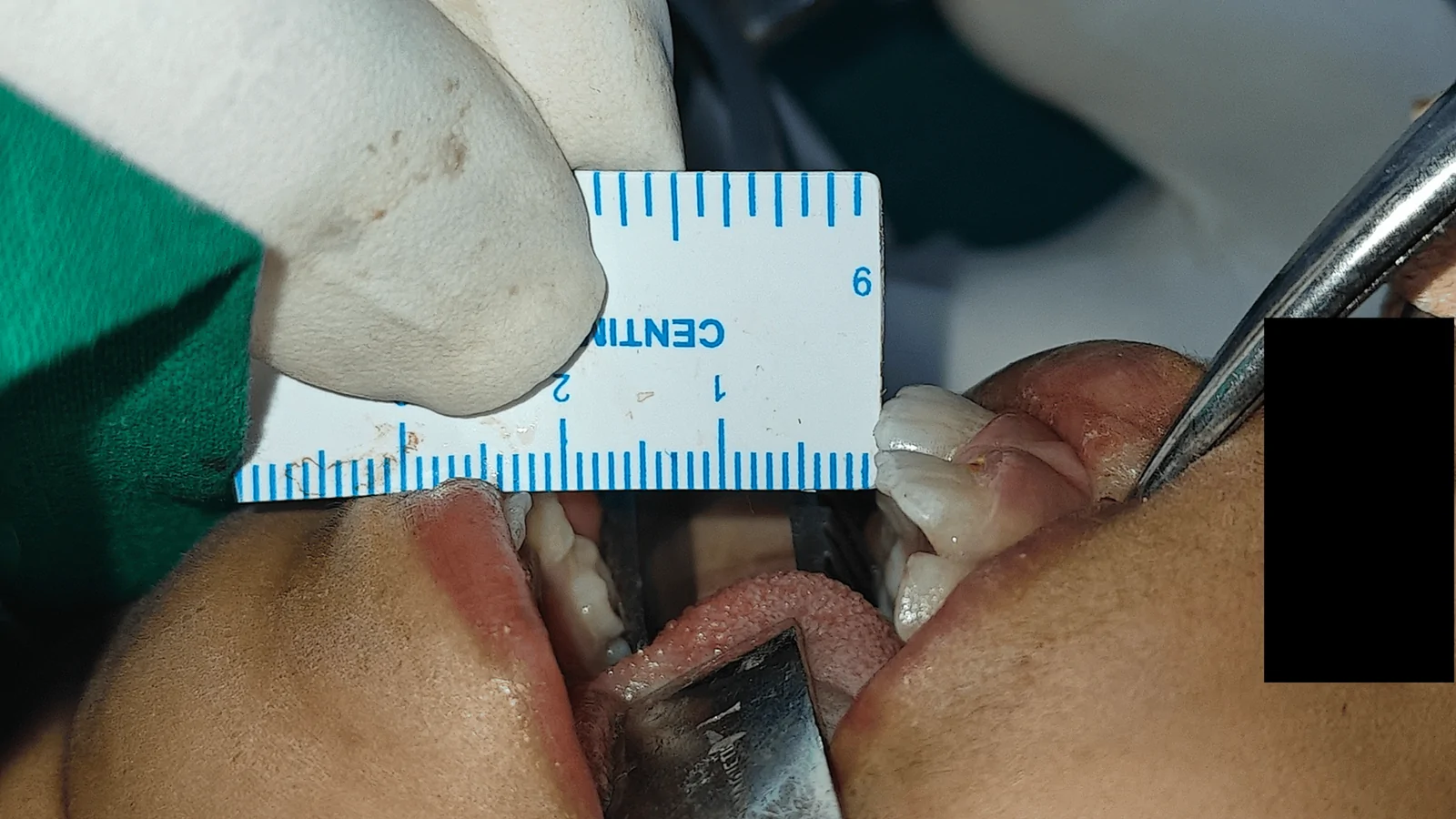
Intraoperative image showing an interincisal mouth opening of 23 mm following gap arthroplasty.

**Figure 6:**
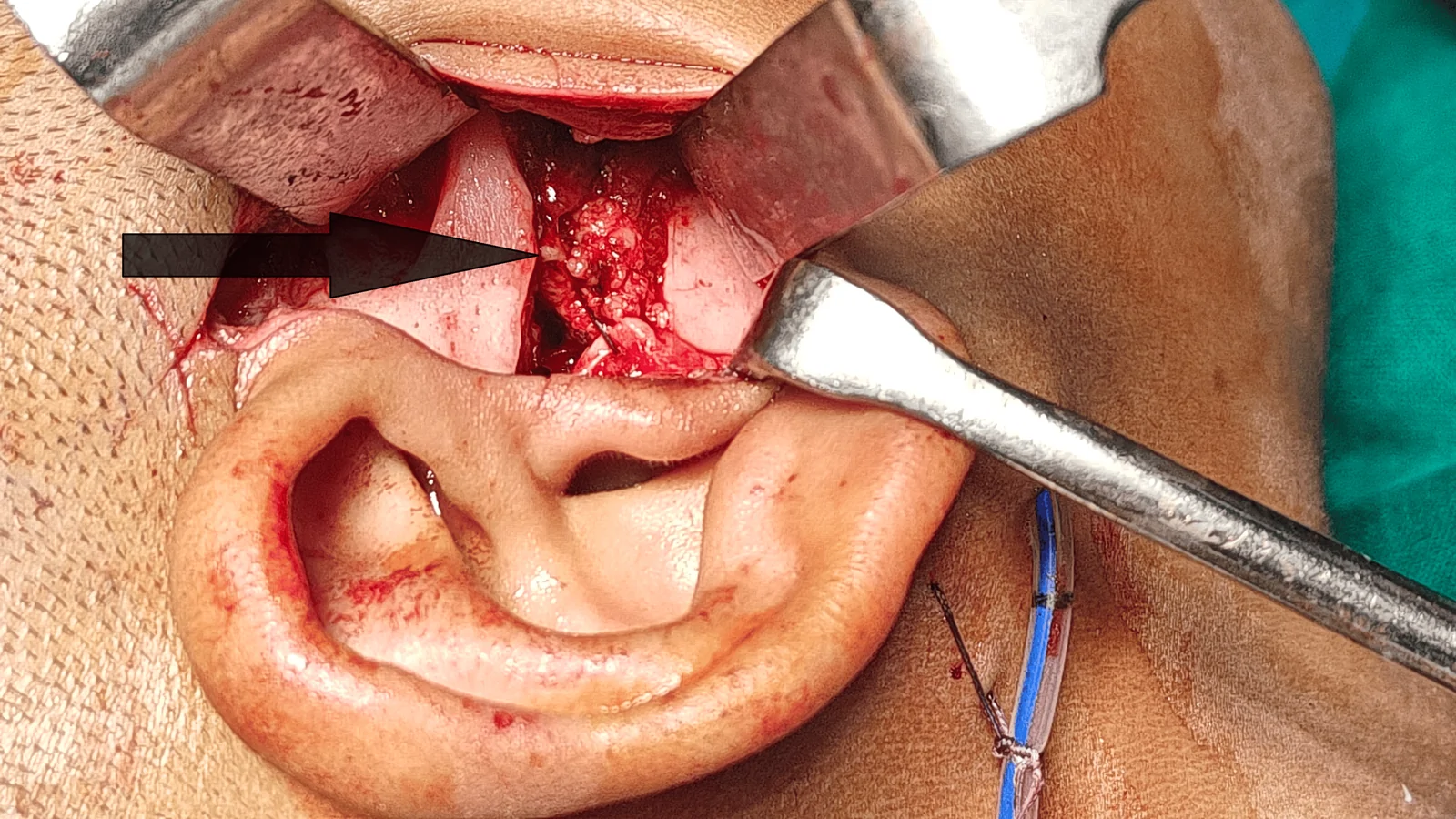
Intraoperative image demonstrating interpositional arthroplasty with placement of a vascularized pedicled buccal fat pad (black arrow).

Approximately 10 mm of gap was created between the glenoid fossa and the mandibular ramus. Ipsilateral coronoidectomy was also performed to eliminate muscular restriction and improve mandibular mobility. Intraoperatively, mouth opening improved to 23 mm (Figure 5). A pedicled buccal fat pad (Figure 6) was used to interpose and obliterate the dead space between the glenoid fossa and the mandibular ramus. It also acted as a biologically compatible cushioning layer between the bony surfaces.

**Figure 7:**
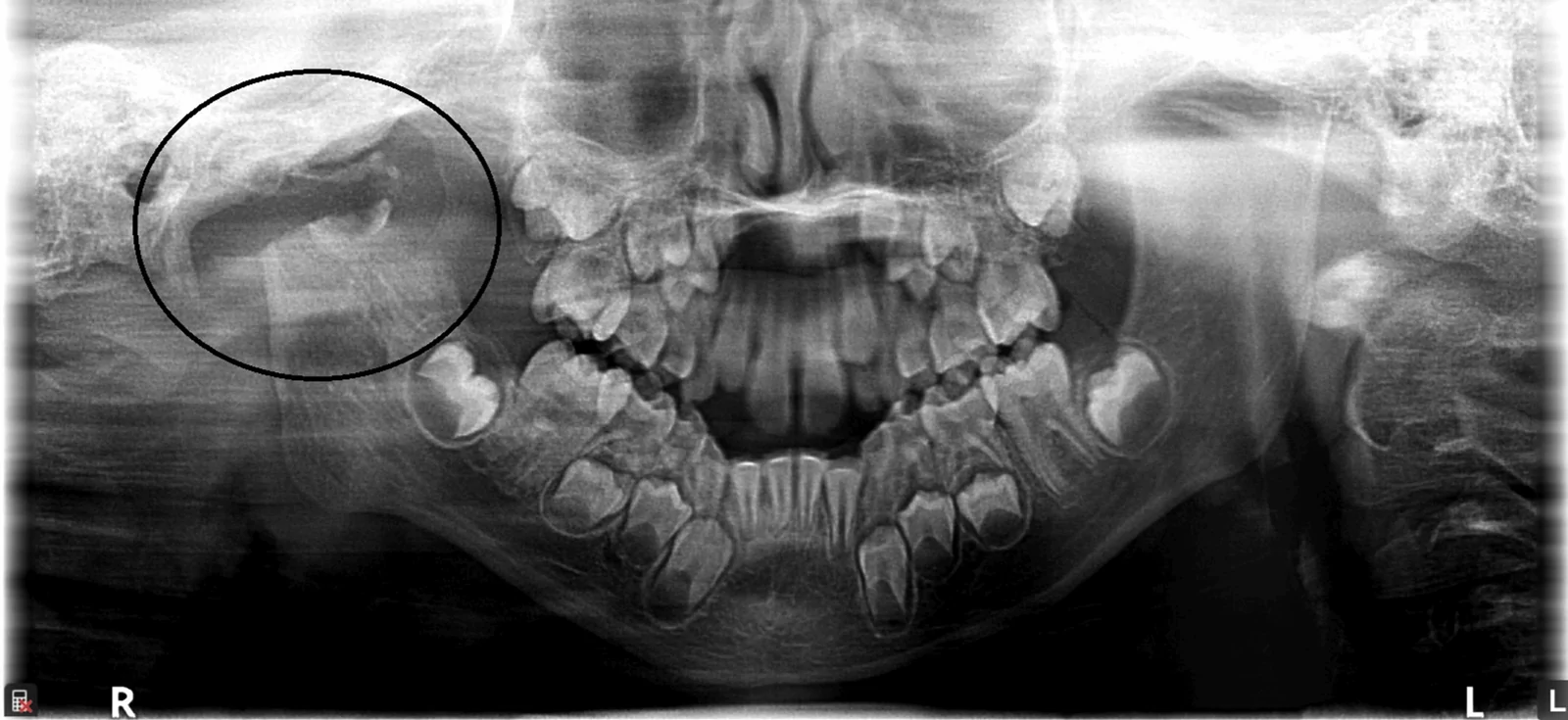
Postoperative orthopantomogram showing the surgically created gap in the right temporomandibular joint (black circle).

The patient was followed up regularly for three months after surgery. A structured physiotherapy program was initiated in the immediate postoperative period, and the patient was instructed to perform regular mouth-opening exercises using stacked ice-cream sticks to maintain the surgically achieved interincisal opening. At the three-month follow-up, the patient was compliant with the physiotherapy regimen and maintained an interincisal mouth opening of approximately 20 mm. Functional improvement was evident, with restoration of adequate mandibular movements, and the patient was able to resume a normal diet without difficulty. No clinical evidence of re-ankylosis or postoperative complications was observed during the follow-up period. OPG revealed a gap created in the right temporomandibular joint space (Figure 7).

## Discussion

TMJa in children requires early surgical intervention to prevent progressive skeletal deformity and functional impairment [1,3]. The present case involved a six-year-old child diagnosed with unilateral Type III bony ankylosis according to Sawhney’s classification. Type III ankylosis is characterized by a bony bridge between the temporal bone and the mandibular ramus with a medially displaced condyle, often requiring aggressive surgical resection for adequate joint release.

Gap arthroplasty remains an established treatment modality; however, recurrence remains a concern [4]. Adequate bone removal and strict postoperative physiotherapy are critical determinants of long-term success. In the present case, a 10-mm gap was created to reduce the likelihood of re-ankylosis.

There are advantages to piezoelectric surgery over rotary osteotomy techniques. Because a selective cutting mechanism leaves adjacent soft tissues uninjured, there is less risk of damaging vital structures such as the facial nerve, internal maxillary artery, and middle cranial fossa [5]. Piezoelectric surgery for TMJa release reduces bleeding and complications, while allowing precise bone cutting. It appears to be a safe and effective alternative to the conventional technique [7].

Piezoelectric surgery is now used for many surgical procedures in oral and maxillofacial surgery, such as dental implants, implant-site preparation, alveolar ridge splitting, bone graft harvesting, lateralization of the inferior alveolar nerve, orthognathic surgery, aesthetic facial surgery, distraction osteogenesis, inferior alveolar nerve preservation, and trauma [8].

Autogenous interpositional materials remain preferable in pediatric patients due to their biological compatibility and adaptability during growth. Vascularized tissues such as the buccal fat pad provide a reliable and well-vascularized interpositional barrier that helps reduce the risk of recurrence [9,10]. It is well established that postoperative physiotherapy plays a key role in preventing re-ankylosis and functional rehabilitation.

## Conclusions

Piezo-assisted gap arthroplasty with vascularized buccal fat pad interposition appeared to provide a favorable functional outcome in this pediatric case of TMJa. However, longer follow-up and larger case series are needed to confirm its safety, effectiveness, and recurrence rate.
